# Trends in Obesity and Metabolic Status in Northern and Southern China Between 2012 and 2020

**DOI:** 10.3389/fnut.2021.811244

**Published:** 2022-01-11

**Authors:** Ying Li, Lin Yang, Lu Yin, Qingqi Liu, Yaqin Wang, Pingting Yang, Jiangang Wang, Zhiheng Chen, Xiaohui Li, Qinyu Yang, Yongmei He, Xin Huang

**Affiliations:** ^1^Department of Health Management, The Third Xiangya Hospital, Central South University, Changsha, China; ^2^Department of Cancer Epidemiology and Prevention Research, Cancer Care Alberta, Alberta Health Services, Calgary, AB, Canada; ^3^Departments of Oncology and Community Health Sciences, Cumming School of Medicine, University of Calgary, Calgary, AB, Canada; ^4^Medical Research & Biometrics Center, National Center for Cardiovascular Diseases, Chinese Academy of Medical Sciences and Peking Union Medical College, Beijing, China; ^5^Department of Biostatistics, Bioinformatics & Biomathematics, Georgetown University, Washington, DC, United States; ^6^Department of Pharmacology, Xiangya School of Pharamceutical Science, Central South University, Changsha, China; ^7^Department of Health Management, Aerospace Center Hospital, Beijing, China; ^8^Department of Epidemiology, School of Medicine, Hunan Normal University, Changsha, China

**Keywords:** obesity, metabolic status, trend, series cross-sectional study, China

## Abstract

**Background:** The trends of obesity-associated metabolic status in Chinese are lacking, especially those from different regions.

**Objectives:** To examine the trends of obesity and metabolic status among Chinese population in 2012–2020.

**Methods:** In a series cross-sectional study, data on 256,782 participants surveyed between 2014 and 2020 in Beijing, northern China, and 697,170 participants surveyed between 2012 and 2020 in Hunan, southern China were analyzed. Anthropometrics, blood pressure measurements, and blood tests were performed according to standard protocols. Trends in obesity and metabolic status were evaluated using the Joinpoint software.

**Results:** Based on age- and sex-standardized values, the mean BMI values in northern and southern participants were 23.94 (95% CI: 23.93, 23.95) and 23.68 (95% CI: 23.67, 23.69) kg/m^2^, respectively. Between 2014 and 2020, the overall obesity prevalence among northern participants increased from 12.70% (95% CI: 12.17, 13.23%) to 14.33% (95% CI: 13.97, 14.70%) (*P* = 0.009), mainly derived by the 20–39 and 40–59 age groups. Moreover, the prevalence of metabolically healthy obese significantly increased from 2.07% (95% CI: 1.84, 2.30%) to 4.33% (95% CI: 4.13, 4.53%) in Northerners. Between 2012 and 2020, no significant trend in obesity was found among overall southern participants, but the prevalence of metabolically unhealthy obese significantly increased from 5.36% (95% CI: 5.18, 5.54%) to 7.35% (95% CI: 7.11, 7.58%), mainly derived by the 20–39 and 40–59 age groups.

**Conclusions:** The trends in obesity and metabolic status were different between southern and northern Chinese. A national weight control plan is needed in China, focusing on young and middle-aged population.

## Introduction

Obesity is a major risk factor for hypertension, diabetes, coronary heart disease, certain types of cancer, and poor mental health (1–5). Approximately 4 million global deaths were due to high body mass index (BMI) in 2015 (6). According to the Global Burden of Disease Study, the worldwide prevalence of overweight and obesity has doubled from 1980 to 2015 (7). With the increasing spread of the global obesity pandemic, China also saw a dramatic increase in overweight and obese adults (8). For example, during 1993–2015, the prevalence of overweight, obesity, and abdominal obesity increased by 14.7, 11.5, and 26.7%, respectively (9). The most recent national nutrition survey during 2015–2019 indicates that obesity was 16.4% in Chinese adults (10). However, the annual trend of change in obesity, especially in different regions, is not available.

According to the metabolic status and the BMI level, the population could be further classified into the following four phenotypes: metabolically healthy non-obese (MHNO), metabolically unhealthy non-obese (MUNO), metabolically healthy obese (MHO), and metabolically unhealthy obese (MUO) (11). Different phenotypes present different cardiovascular and metabolic complications risks (11–14). However, the data on trends in obesity-related metabolic status in the Chinese population are lacking.

Moreover, the coronavirus disease 2019 (COVID-19) pandemic has caused significant disruption in everyday lifestyle since 2020. The previous study indicated that the COVID-19 pandemic has serious consequences for the obesity epidemic. And in turn, obesity and impaired metabolic health also emerged as important determinants of severe COVID-19 (15). The changes in body weight and metabolic status that occurred during the COVID-19 pandemic in China are unknown.

The current study provides new estimates of the prevalence of obesity and its metabolic status in large populations from two southern and northern areas in China.

## Patients and Methods

### Study Population

The serial cross-sectional study population comprised more than 900,000 individuals from a mixed urban and rural area who visited health management centers in Beijing and Hunan, two northern and southern regions in China, between 2012 and 2020. The overall population of Hunan is over 60 million, and that of Beijing is around 20 million. In the present study, ~80% of the participants in Hunan were from urban areas, while 95% of the Beijing area was urban. Participants with diverse socioeconomic background (public services employees, workers, self-employed persons, farmers, and others) came to health management centers to check their health status was enrolled in the current study. All participants signed informed consent forms, and the Ethics Committee of the Third Xiangya Hospital approved the study (2020-S498).

**Figure 1:**
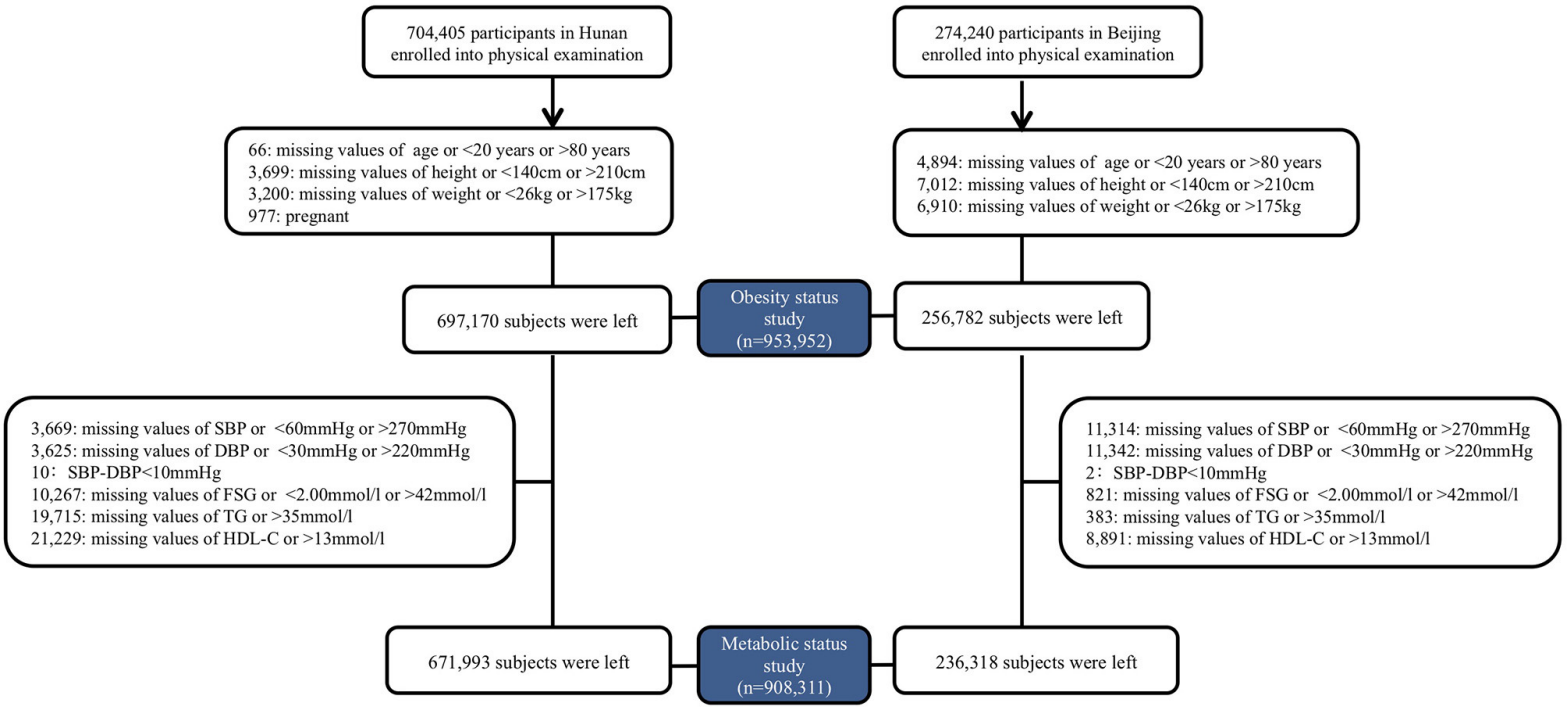
Enrollment flowchart.

All enrolled participants underwent a routine clinical examination. Participants recorded age, sex, current medication use, and previous medical diagnoses by themself in Beijing, northern China. Beginning in 2018, physicians reconfirmed the questionnaires during the physical examination. More detailed questionnaires, including exercise, smoking history, alcohol consumption, and food consumption, were obtained and checked by physicians in Hunan, southern China (16). Individuals with missing data or unreasonable values on age (<20 or >80 years), height (<140 or >210 cm), or weight (<26 or >175 kg) were excluded. Participants with missing or unreasonable blood pressure [Systolic Blood Pressure (SBP) <60 or >270 mmHg; Diastolic Blood Pressure (DBP) <30 or >220 mmHg; Pulse Pressure (PP) <10 mmHg], Fasting Serum Glucose (FSG) (<2.00 or >42 mmol/l) or lipids [Triglyceride (TG) >35 mmol/l; Total Cholesterol (TC) >20 mmol/l; Low-Density Lipoprotein cholesterol (LDL-c) >15 mmol/l; High-Density Lipoprotein cholesterol (HDL-c) >13 mmol/l] were further excluded from metabolically status classification. The enrollment process was listed in Figure 1. Assessment methods are detailed in the Supplementary Material.

### Measurement and Definition

Physical examinations were conducted with the same methods described in our previous study (17). Briefly, blood pressure, height, and weight were measured by trained physicians. Participants were categorized into non-obese (<28 kg/m^2^) and obese (≥28 kg/m^2^) groups (18). Moreover, participants who met two or more of the following four criteria were considered metabolically unhealthy: high TG (≥1.7 mmol/L) or using lipid-lowering drugs, elevated SBP (≥130 mmHg) or DBP (≥85 mmHg) or using anti-hypertensive drugs, high FSG (≥5.6 mmol/L) or using medications for diabetes (insulin and oral anti-diabetic), and low HDL-c (<1.04 mmol/L for men and <1.29 mmol/L for women) (11). Then the participants were furtherly classified into the MHNO MUNO, MHO, and MUO phenotypes. Definition of chronic diseases are detailed in the Supplementary Material.

### Laboratory Measurements

Fasting venous blood samples were collected and were immediately processed and analyzed at the clinical laboratory of Third Xiangya Hospital (in Hunan) or Aerospace Center Hospital (in Beijing), as detailed in the Supplementary Material.

### Statistical Analysis

Continuous variables were expressed as means with 95% confidence intervals (95% CIs), categorical variables were expressed as percentages with 95% CIs, and differences among different BMI groups were tested by analysis of variance and the Chi-square test. Restricted cubic spline regression models were used to test the overall and non-linear association between survey years and BMI, HDL-c, TG, SBP, DBP, and FSG. Then, the linear association would be evaluated when the test for non-linearity is not significant. Means of BMI and metabolic status were further stratified by location and sex because of significant 3-way interactions. Trends in the prevalence of obesity and metabolic subtypes were evaluated using the Joinpoint Regression Program (Version 4.9.0.0) (19), and annual percentage changes (APCs) in slopes were reported. Moreover, the prevalence of obesity and metabolic subtypes were stratified by location, age group (20–39, 40–59, 60, and older years), and sex because of significant 4-way interactions. The prevalence of obesity, MHNO, MUNO, MHO, and MUO and the mean levels of BMI, TC, HDL-c, and TG, SBP, DBP, and FSG were all estimated for females and males after age standardization according to the population distribution in China in 2010 (20). The weights were 0.4385, 0.3860, and 0.1755 for the 20–39, 40–59, and over 60 years old groups, respectively. When calculating estimates for each survey year, sex was additionally adjusted, and weights for females and males were the same as 0.5. A two-sided *P* < 0.05 was considered to be statistically significant. SAS version 9.4 (SAS Institute Inc) was used for analyses.

## Results

### Characteristics of Selected Study Participants

A total of 256,782 participants were surveyed from January 1, 2014, to December 31, 2020, in northern China, and 697,170 participants were surveyed from January 1, 2012, to December 31, 2020, in southern China (Figure 1). Overall, the mean BMI was 23.91 (95% CI: 23.90, 23.92) kg/m^2^ and 108,914 participants [11.41% (95% CI: 11.34, 11.47%)] were obese. Table 1 shows the demographic and clinical characteristics of the study participants by obesity status. Compared to the non-obese, participants in the obese group were more likely to be male, from northerners, older, with a higher level of TC, TC, LDL-c, and FSG, having elevated blood pressure, with hypertension, diabetes, or hyperlipidemia (*P* < 0.001, Table 1).

**Table 1:** Characteristics of selected study participants by obesity status.

**Characteristics**		**Obesity** ***N*** **(%) /mean (SD)**	**Non-obesity** ***N*** **(%) /mean (SD)**
Location**	Northern China	35,953 (33.01)	220,829 (26.13)
	Southern China	72,961 (66.99)	624,209 (73.87)
Sex**	Female	23,584 (21.65)	386,537 (45.74)
	Male	85,330 (78.35)	458,501 (54.26)
Age**, year (*N* = 953,952)		46.13 (14.20)	44.56 (14.79)
BMI, kg/m^2^ (*N* = 953,952)		30.10 (2.10)	23.11 (2.65)
TG**, mmol/L (*N* = 933,854)		2.46 (2.15)	1.53 (1.39)
TC**, mmol/L (*N* = 933,872)		5.14 (1.01)	4.90 (0.95)
HDL-c**, mmol/L (*N* = 923,832)		1.15 (0.29)	1.39 (0.39)
LDL-c**, mmol/L (*N* = 923,715)		2.84 (0.85)	2.72 (0.80)
SBP**, mmHg (*N* = 938,969)		132.81 (16.08)	121.36 (16.31)
DBP**, mmHg (*N* = 938,985)		83.27 (11.57)	74.79 (10.92)
FSG**, mmol/L (*N* = 942,864)		5.84 (1.61)	5.35 (1.18)
Hypertension**	No	62,506 (58.53)	681,807 (81.93)
	Yes	44,278 (41.47)	150,342 (18.07)
Diabetes**	No	94,512 (87.50)	791,011 (94.75)
	Yes	13,500 (12.50)	43,841 (5.25)
Dyslipidemia**	No	40,345 (38.01)	546,177 (66.82)
	Yes	65,785 (61.99)	271,260 (33.18)

** *p <0.01*.

*SD, standard deviation; BMI, body mass index; SBP, systolic blood pressure; DBP, diastolic blood pressure; FSG, fasting serum glucose; TG, triglyceride; TC, total cholesterol; LDL-c, low-density lipoprotein cholesterol; HDL-c, high-density lipoprotein cholesterol*.

*Obesity was defined as BMI ≥ 28 kg/m^2^*.

*Hypertension was defined as self-reported hypertension diagnosed by a physician, self-reported regular use of antihypertensive medications, or systolic/diastolic blood pressure at recruitment ≥ 140/90 mmHg*.

*Dyslipidemia was defined as meeting any of the following criteria: (1) TC ≥ 6.22 mmol/L; (2) LDL-C ≥ 4.14 mmol/L; (3) HDL-C <1.04 mmol/L; (4) TG ≥ 2.26 mmol/L; (5) self-reported dyslipidemia or use of lipid-lowering medications; Diabetes mellitus was defined as self-reported diabetes diagnosed by a physician, self-reported regular use of antidiabetic medications, or fasting glucose at recruitment ≥ 7.0 mmol/L*.

### Trends in BMI and Obesity

**Figure 2:**
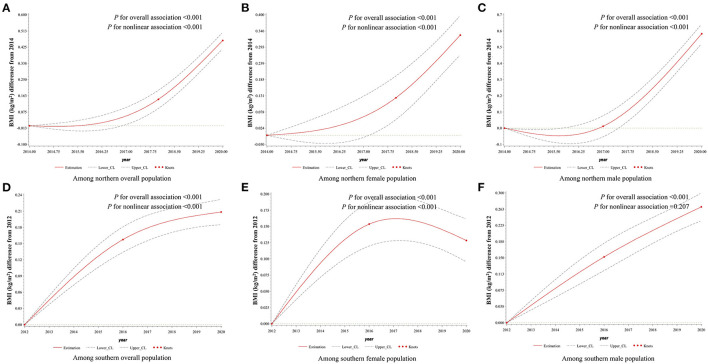
Association between survey years and BMI among adults aged 20 years and older in northern and southern China, 2012–2020. Surveyed year was coded using the RCS function with three knots located at the 5th, 50th, and 95th percentiles of the distribution of survey years. Y-axis represents the BMI difference from referenced year. Referenced year in **(A–C)** was 2014, and in **(D–F)** was 2012. Dashed lines are 95 percent confidence intervals. Knots are represented by dots. In **(A)** and **(D)**, age and sex were included as adjustment variables. In **(B,C,E,F)**, age was included as an adjustment variable. BMI, body mass index.

The age- and sex-standardized mean BMI levels between northerners and southerners were 23.94 (95% CI: 23.93, 23.95) and 23.68 (95% CI: 23.67, 23.69) kg/m^2^, respectively (Table 2). During 2014–2020, the BMI levels in northerners showed significant non-linear changes by surveyed year (*P* < 0.001) and grew rapidly after 2018 (Figure 2). A non-linear trend in BMI level among female from south was also observed, which increased from 2012 to 2016 and declined after 2016. However, a significant and linear increasing trend in BMI trend was observed among the southern male population (*P* < 0.001; Figure 2). Due to the interaction between BMI and age, we further stratified the population by age. As a result, the BMI level was found to significantly increase among 20–39 aged groups in both areas, and the trends between southern male and northern female groups were linear. Among the 40–59 years old, non-linear upward trends were observed in both sexes from southern and northern China. On the other hand, a significant and downward trend in BMI was found among female participants over 60 from southern China (Supplementary Table 1, Supplementary Figure 1).

**Table 2:** Age-Standardized mean and 95% confidence interval of BMI levels among adults aged 20 years and older in northern and southern China, 2012–2020^*^.

**Year**	**Northern China BMI, kg/m^2^ [mean (95% CI)]**			**Southern China BMI, kg/m^2^ [mean (95% CI)]**		
	**All^$^**	**Female**	**Male**	**All^$^**	**Female**	**Male**
2012				23.46 (23.44, 23.49)	22.33 (22.30, 22.37)	24.59 (24.56, 24.62)
2013				23.63 (23.61, 23.66)	22.46 (22.43, 22.49)	24.80 (24.78, 24.83)
2014	23.85 (23.80, 23.90)	22.76 (22.69, 22.84)	24.94 (24.87, 25.00)	23.74 (23.72, 23.76)	22.60 (22.57, 22.63)	24.88 (24.85, 24.91)
2015	23.67 (23.63, 23.71)	22.59 (22.54, 22.65)	24.75 (24.70, 24.80)	23.75 (23.73, 23.77)	22.59 (22.56, 22.62)	24.90 (24.87, 24.93)
2016	23.78 (23.74, 23.82)	22.71 (22.66, 22.76)	24.85 (24.80, 24.90)	23.61 (23.58, 23.63)	22.46 (22.43, 22.49)	24.75 (24.72, 24.78)
2017	23.78 (23.75, 23.82)	22.68 (22.63, 22.73)	24.89 (24.85, 24.93)	23.68 (23.66, 23.70)	22.54 (22.51, 22.57)	24.82 (24.79, 24.85)
2018	23.97 (23.94, 24.00)	22.92 (22.87, 22.96)	25.02 (24.98, 25.06)	23.67 (23.65, 23.70)	22.46 (22.43, 22.49)	24.89 (24.86, 24.92)
2019	24.04 (24.02, 24.07)	22.99 (22.95, 23.03)	25.10 (25.06, 25.13)	23.79 (23.77, 23.81)	22.60 (22.57, 22.63)	24.98 (24.95, 25.01)
2020	24.16 (24.13, 24.19)	22.98 (22.94, 23.03)	25.34 (25.29, 25.38)	23.73 (23.70, 23.75)	22.49 (22.45, 22.53)	24.96 (24.93, 25.00)
*p* for overall trend	<0.0001	<0.0001	<0.0001	<0.0001	<0.0001	<0.0001^#^
*p* for non-linear trend	<0.0001	<0.0001	<0.0001	<0.0001	<0.0001	0.207
Subtotal	23.94 (23.93, 23.95)	22.86 (22.84, 22.88)	25.02 (25.01, 25.04)	23.68 (23.67, 23.69)	22.51 (22.50, 22.53)	24.84 (24.83, 24.85)

* *Estimates are age-standardized to the 2010 Chinese Census population using age groups 20–39, 40–59, and 60 or older; $ additional adjusted by sex; #P for linear trends < 0.001*.

**Figure 3:**
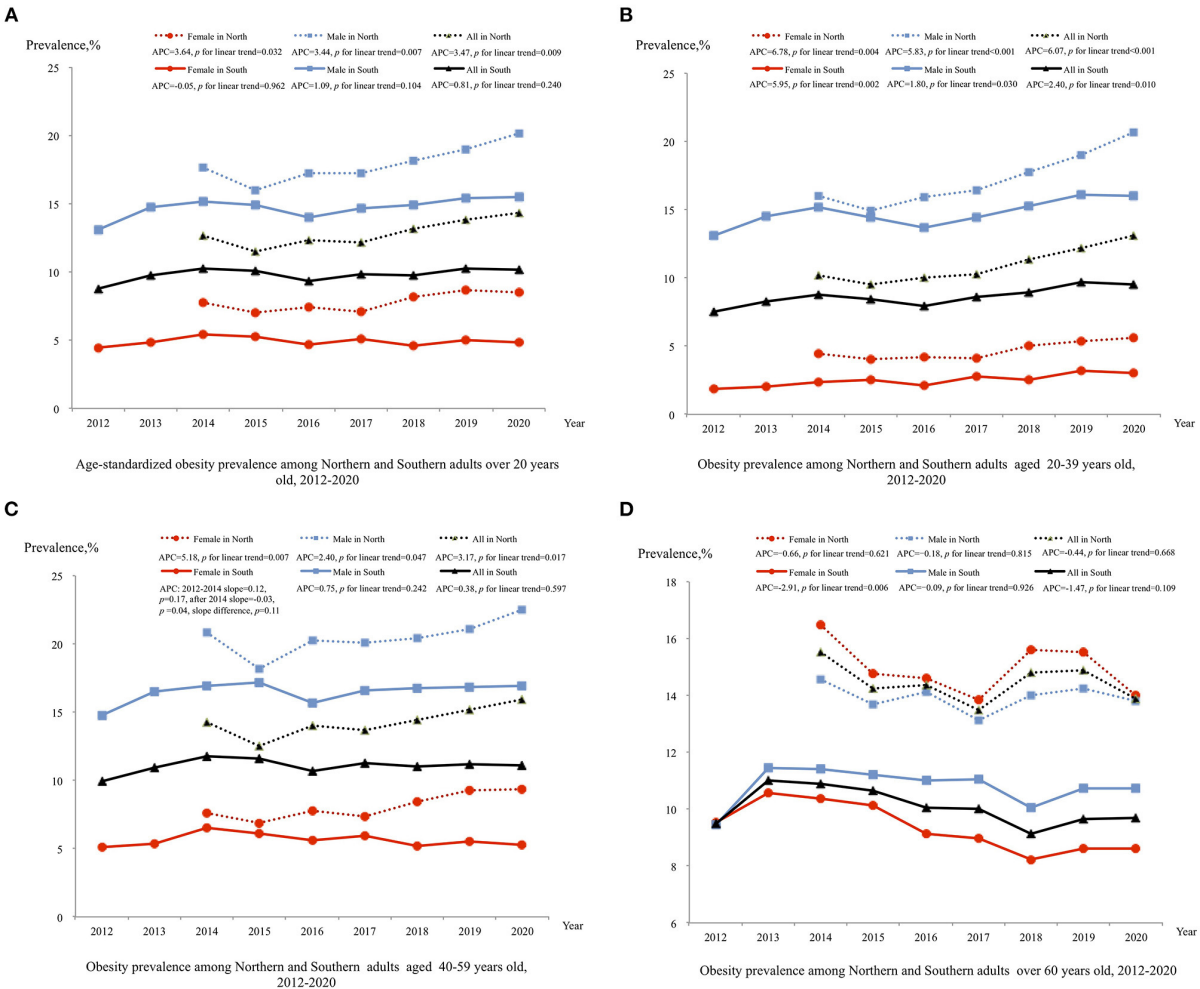
Trend in prevalence of obesity adults in northern and southern China, 2012–2020. Estimates by different sex are age-standardized to the 2010 Chinese Census population using age groups 20–39, 40–59, and 60 or older and estimates for overall population are additional adjusted by sex; Significant linear trends (*P* < 0.05) for the following groups:(1) increased age-standardized obesity among all sex groups from northern China; (2) increased obesity among all 20–39 aged groups from northern and southern China; (3) increased obesity among all 40–59 aged groups from northern (*P* < 0.05); (4) among 40–59 aged female from southern China, obesity during 2012–2014 showed increased level [slope = 0.12 (95% CI: −0.07,0.30), *P* = 0.17], and level decreased after 2014 [slope = −0.03 (95% CI: −0.06, 0.00, *P* = 0.04], slope difference, *P* = 0.11; (5) decreased obesity among 60 and over aged female from southern. BMI, body mass index; APC, annual percentage change.

Between 2014 and 2020, the prevalence of obesity among northerners increased from 12.70% (95% CI: 12.17, 13.23%) to 14.33% (95% CI: 13.97, 14.70%) (*P* = 0.009; Figure 3A). The obesity prevalence among northern females and males showed similar annual percentage changes. Among the 20–39 and 40–59 age groups, the APCs were 6.07 (95% CI: 3.97, 8.21) and 3.17 (95% CI: 1.02, 5.37), respectively. However, the trends of obesity in the over 60 years age group were non-significant (*P* = 0.668; Figures 3B–D).

The trends of obesity among southerners were different from those among northerners. Between 2012 and 2020, no significant trend in obesity was found among the total sample of southerners (*P* = 0.240; Figure 3A). After stratification by age and sex, the prevalence of obesity among females and males aged 20–39 years was found to increase from 1.87% (95% CI, 1.63, 2.11%) to 2.99% (95% CI, 2.69, 3.29%) and from 13.10% (95% CI, 12.53, 13.67%) to 16.04% (95% CI, 15.34, 16.74%), respectively (Figure 3B). However, obesity among over 60 years old southern females showed significant decreasing trends (*P* < 0.006), and among 40–59 years aged southern females, the prevalence of obesity increased between 2012 and 2014 [slope = 0.12 (95% CI: −0.07,0.30), *P* = 0.17], and decreased after 2014 [slope = −0.03 (95% CI: −0.06, 0.00), *P* = 0.04; Figures 3C,D].

### Trends in Metabolic Factors

Overall, a total of 908,311 persons were included for metabolic analysis. The age-adjusted means of FSG, TG, HDL-c, SBP, and DBP are listed in Supplementary Tables 2–6. Overall, the changing trend in metabolic factors was similar between different sexes but showed regional differences in FSG, which were both rising in the two regions (Supplementary Figure 2). The TG levels showed a downward trend in 2014-2018 and an upward trend in 2018–2020 among the northern population, and in the south, it showed a non-linear monotonous rising trend (Supplementary Figure 3). The HDL-c levels had opposite trends in the two places, with northern rising and southern declining (Supplementary Figure 4). The SBP levels showed an upward trend in the north, and in the south, it showed a downward trend in 2012–2016 and an upward trend in 2016–2020 (Supplementary Figure 5). The DBP levels rose first and then fell in the north, and fell first and then rose in the south (Supplementary Figure 6).

### Trends of Obesity Phenotypes

**Figure 4:**
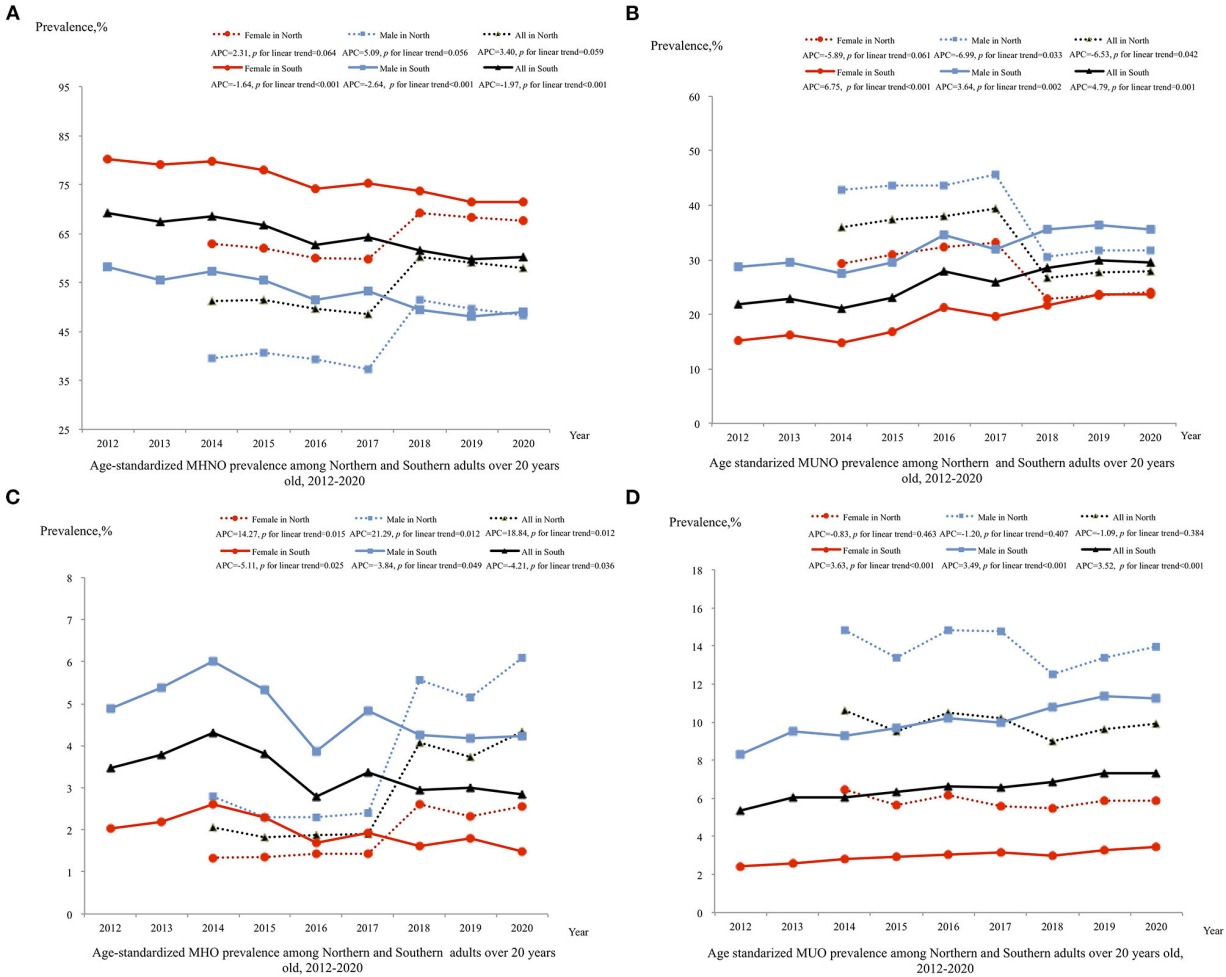
Trend in prevalence of obesity by metabolic status phenotype among adults in northern and southern China, 2012–2020. Estimates by different sex are age-standardized to the 2010 Chinese Census population using age groups 20–39, 40–59, and 60 or older and estimates for overall population are additionally adjusted by sex; Significant linear trends (*P* < 0.05) for the following groups: (1) decreased MHNO among all sex groups from southern; (2) decreased MUNO among male and overall groups from northern (*P* < 0.05); increased MUNO among all sex groups from southern (*P* < 0.05); (3) increased MHO among all sex groups from northern and decreased MHO among all sex groups from southern; (4) increased MUO among all sex groups from southern (*P* < 0.05). MHNO, metabolic healthy non-obese; MUNO, metabolic unhealthy non-obese; MHO, metabolic healthy obese; MUO, metabolic unhealthy obese; APC, annual percentage change.

The trends of obesity phenotypes have been listed in Figures 4A–D. Between 2014 and 2020, among the northern participants, the prevalence of the MUNO subtype significantly decreased from 36.03% (95% CI: 35.16, 36.90%) to 27.85% (95% CI: 27.31, 28.39%) with an annual percentage change of −6.53 (95% CI: −12.33, −0.34) (Figure 4B), but the trends in MUO were non-significant (*P* = 0.384; Figure 4D). However, the trends in MHNO were non-significant (Figure 4A), and the prevalence of MHO increased significantly from 2.07% (95% CI: 1.84, 2.30%) to 4.33% (95% CI: 4.13, 4.53%) (Figure 4C). All obesity phenotypes in different sex and age groups among northerners showed similar annual percentage changes (Supplementary Figure 7).

Unlike the trends in obesity phenotypes among northerners, between 2012 and 2020, the prevalence of the metabolically unhealthy subtypes MUNO and MUO among southerners increased significantly from 21.92% (95% CI: 21.55, 22.30%) to 29.58% (95% CI: 29.10, 30.07%) and from 5.36% (95% CI: 5.18, 5.54%) to 7.35% (95% CI: 7.11, 7.58%), respectively (Figures 4B,D). Meanwhile, the prevalence of the metabolically healthy subtypes MHNO and MHO decreased significantly from 69.24% (95% CI: 68.57, 69.92%) to 60.21% (95% CI: 59.55, 60.88%) and from 3.47% (95% CI: 3.32, 3.61%) to 2.86% (95% CI: 2.71, 3.00%), respectively (Figures 4A,C). Among southerners aged 40–59 and over 60 years old, females and males showed a similar annual percentage change in all obesity phenotypes (Supplementary Figure 7), but among the 20–39 year age group, the prevalence of MUNO and MUO in females showed a greater annual percentage increase than that in males [14.84 (95% CI: 9.40, 20.56) vs. 5.18 (95% CI: 2.62, 7.81) and 15.02 (95% CI: 13.55, 16.51) vs. 5.32 (95% CI: 3.74, 6.92)] (Supplementary Figure 7).

## Discussion

The present analyses show that the mean BMI and the prevalence of obesity in northern China were significantly higher than in southern China. However, compared to the north, marked changes in metabolic abnormalities occurred in the south from 2012 to 2020. The mean BMI and the prevalence of obesity increased significantly in the north, driven primarily by 20–39- and 40–59-years adult males and females from 2014 to 2020. In the south, the mean BMI increased significantly, but the overall obesity prevalence was stable. Moreover, the proportion of MUO increased, especially in 20–39 years females, and 20–39- and 40–59-years male population.

In a previous study, the age-standardized mean BMI increased by 2.0 kg/m^2^ from 1993 to 2015 in China (9). Our study further confirms that the mean BMI continued to grow in both areas from 2012 to 2020. But the trend in BMI differed between northern and southern China. The mean BMI level in northern China increased rapidly after 2018, whereas in southern China, it increased obviously from 2012 to 2016. Moreover, unlike the other study (9), the increased mean BMI level was mainly derived by young and middle-aged population, especially in the north.

The trend in obesity prevalence in our study was inconsistent with other studies. Globally, the prevalence of obesity increased from 5% in 1980 to 10.1% in males and from 8.9 to 14.8% in females in 2015 (21). Based on cross-sectional surveys, the prevalence of obesity increased from 4.2 to 15.7% in the Chinese population between 1993 and 2015 (9). In the other regional studies, the prevalence of obesity in Jilin and Nanjing, two cities located in eastern China increased by 3.6 and 4.0% for males, and by 5.6 and 1.5% for females from 2007 to 2013 and 2008 to 2016 respectively (22). However, the increase in obesity in northern was 3.47% per year, while the current study found no significant increasing trend in Hunan, southern China. In Wang's study, the change in the prevalence of obesity in Liangshan Yi Autonomous Prefecture migrants was −0.6% in Sichuan, western China, from 2007 to 2015 (23). The geographical position of Hunan Province borders Sichuan Province; thus, the demographic characteristics might be similar between those two areas.

In addition, aged 20–39 and 40–59 populations drove the increasing trend of the prevalence of obesity in Beijing. The turning point of the prevalence of obesity in females aged 40–59 and the declining trend in females over 60 years old have evened out the rising rate in both females and males aged 20–39. Thus, the overall obesity rate did not show an increasing trend in southern China. In previous studies, the increasing trend in obesity was driven by all age subgroups, especially by the 40–80 aged population surveyed before 2015 (24). Our study indicated that more attention should be given to the young and middle-aged populations in China.

It is demonstrated that the COVID-19 pandemic could have serious consequences for the obesity epidemic (15). A study conducted in Italy has shown that home isolation and adverse mental health burden linked to the COVID-19 pandemic were associated with significant weight gain in 2020 (25). In the current study, neither the mean BMI nor the prevalence of the obesity rate showed a significant change in 2020 that deviated from the previous trend. In our view, the rapidly controlled COVID-19 pandemic might not have had significantly impact on the weight in China. Some previous reports of weight gain during the pandemic might have obtained significant results because they did not compare to previous trends.

In our study, the prevalence of MHO was 4.33 and 2.95% in northern and southern China in 2020, respectively. In the China Kadoorie Biobank study, the MHO phenotype accounted for 3.3% of the total population from 2004 to 2008 in China (26). According to a previous study, the prevalence of MHO has been shown to range between 4.2 and 13.6% in a random sample from a Chinese adult population, depending on the definition used for MHO (27). The trends in the prevalence of obesity metabolic status differed between northern and southern China. Overall, the increasing trend in the prevalence of obesity was predominantly driven by MHO in all age groups in the north. During the period of 1973–1980, Keyes and Reuben Andres suggested that MHO could be benign and not contribute to cardiovascular risk (28). However, an increasing number of studies have demonstrated MHO is indeed associated with an increased risk of cardiovascular disease, chronic kidney disease, non-alcoholic fatty liver disease, and death (29–32). Therefore, MHO could be a risk factor for chronic disease (33) and finally transit to MUO (26) if timely intervention is not performed in northern China. In support, although the overall prevalence of obesity was stable, the prevalence of MUO phenotype has increased, while the prevalence of MHO has decreased, especially in young and middle-aged groups in southern China. A previous study done in Shanghai (eastern China) adults indicated the prevalence of metabolism problem was doubled with an increase in metabolically unhealthy overweight from 2002 to 2017 as well (34). There is no doubt that MUO has the most significant impact on health. Therefore, residents in southern China should pay more attention to metabolic status.

High energy intake, especially sugary drinks, and other energy-dense foods, and low levels of physical activity contributed to the increasing trend of obesity in China (35, 36). In the current study, the relatively slow growth trend in the north and the stable trend in the south may be due to the following reasons: First, recognizing the immediacy of chronic disease challenges, the “China Healthy Lifestyle for All” initiative launched in 2007 was developed to raise awareness of a range of preventive health issues, such as knowledge of dietary guidelines, and the adoption of health-promoting behaviors. Currently, much evidence suggests a positive role in healthy lifestyle action after 2015 (37). Thus, good knowledge of healthy lifestyles may help control weight in Chinese adults. Second, the current study participants were from annual health check-up population who received health education and guidance on weight intervention from the Health Management Center. Therefore, the result may not be representative of the data of the national epidemiologic survey.

Our study has several strengths. First, it included a large sample size (>900,000 participants) from Beijing and Hunan, two regions of northern and southern China. Second, the surveys were performed every year between 2012 and 2020 to facilitate the analyses for annual trend of change. Third, we also used physical examination data to analyze the metabolic status and further understand the types of obesity. However, several limitations should be considered. First, only health check-up subjects were investigated in two institutions of health management centers, who were community-derived but not represent random samples. Hence, our results may not be generalizable to overall Chinese population due to selection bias. Second, BMI could not differentiate fat from lean mass or consider the distribution of adipose tissue, while waist circumference (WC), hip circumstance (HC), and waist-to-hip ratio (WHR), which focus on abdominal adiposity, have been identified as useful weight-related anthropometric measures to predict the risk of chronic disease (38). But unfortunately, waist and hip circumferences were not regular measurements in our northern participants. Thus, WC and HC were not analyzed in the current study. Third, the questionnaires were obtained from participants under voluntary principle before 2018 in Beijing. Therefore, some information such as medication use history may be lower than the real-world data. Fourth, lifestyle was associated with obesity and metabolism disorder (39). But we were unable to examine the roles of nutrition and lifestyle factors (e.g., physical activity and sleep duration) on obesity trends because these data were not continuously collected in northerners.

## Conclusions

The trends in the prevalence of obesity and metabolic status were different between northern and southern Chinese. The northerners were dominated by the growth of young and middle-aged obesity and MHO phenotype, while although the overall obesity rate was stable in the south, the proportion of MUO increased, especially in the young and middle-aged population. The weight control plan should generalize to the young and middle-aged Chinese.

## Data Availability Statement

The raw data supporting the conclusions of this article will be made available by the authors, without undue reservation.

## Ethics Statement

The studies involving human participants were reviewed and approved by Ethics Committee of the Third Xiangya Hospital. The patients/participants provided their written informed consent to participate in this study.

## Author Contributions

YL, LYa, LYi, QL, and XH produced data for analysis. YL, LYa, XH, and YH wrote the manuscript. YL, XH, and YH designed the study and handled funding and supervision. PY, YW, JW, ZC, XL, QY, and YH included patients for the study. All authors reviewed and edited the manuscript, read, and approved the final manuscript.

## Funding

This work was supported by funding from the National Natural Science Foundation of China (81973324 to YL, 81872685 to XH, and 82003537 to XH), Hunan Young Talent grant (2020RC3063 to YL), Natural Science Foundation of Hunan Province (2020JJ5858 to YL, 2020JJ4439 to XH), and the Wisdom Accumulation and Talent Cultivation Project of the Third XiangYa hospital of Central South University (YX202002 to YL). The funders had no role in study design, data collection and analysis, decision to publish, or preparation of the manuscript.

## Conflict of Interest

The authors declare that the research was conducted in the absence of any commercial or financial relationships that could be construed as a potential conflict of interest.

## Publisher's Note

All claims expressed in this article are solely those of the authors and do not necessarily represent those of their affiliated organizations, or those of the publisher, the editors and the reviewers. Any product that may be evaluated in this article, or claim that may be made by its manufacturer, is not guaranteed or endorsed by the publisher.
