# Corrigendum: Trends in Obesity and Metabolic Status in Northern and Southern China Between 2012 and 2020

**DOI:** 10.3389/fnut.2022.872237

**Published:** 2022-03-08

**Authors:** Ying Li, Lin Yang, Lu Yin, Qingqi Liu, Yaqin Wang, Pingting Yang, Jiangang Wang, Zhiheng Chen, Xiaohui Li, Qinyu Yang, Yongmei He, Xin Huang

**Affiliations:** ^1^Department of Health Management, The Third Xiangya Hospital, Central South University, Changsha, China; ^2^Department of Cancer Epidemiology and Prevention Research, Cancer Care Alberta, Alberta Health Services, Calgary, AB, Canada; ^3^Departments of Oncology and Community Health Sciences, Cumming School of Medicine, University of Calgary, Calgary, AB, Canada; ^4^Medical Research & Biometrics Center, National Center for Cardiovascular Diseases, Chinese Academy of Medical Sciences and Peking Union Medical College, Beijing, China; ^5^Department of Biostatistics, Bioinformatics & Biomathematics, Georgetown University, Washington, DC, United States; ^6^Department of Pharmacology, Xiangya School of Pharamceutical Science, Central South University, Changsha, China; ^7^Department of Health Management, Aerospace Center Hospital, Beijing, China; ^8^Department of Epidemiology, School of Medicine, Hunan Normal University, Changsha, China

**Keywords:** obesity, metabolic status, trend, series cross-sectional study, China

In the original article, there were some mistakes in Table 1 as published. The means and SD of TG, TC, HDL-c, LDL-c, SBP, DBP and FSG in the non-obesity group were incorrect. The corrected Table 1 appears below.

**Table 1:** Characteristics of selected study participants by obesity status.

**Characteristics**		**Obesity** ***N*** **(%) /mean (SD)**	**Non-obesity** ***N*** **(%) /mean (SD)**
Location**	Northern China	35,953 (33.01)	220,829 (26.13)
	Southern China	72,961 (66.99)	624,209 (73.87)
Sex**	Female	23,584 (21.65)	386,537 (45.74)
	Male	85,330 (78.35)	458,501 (54.26)
Age**, year (*N* = 953,952)		46.13 (14.20)	44.56 (14.79)
BMI, kg/m^2^ (*N* = 953,952)		30.10 (2.10)	23.11 (2.65)
TG**, mmol/L (*N* = 933,854)		2.46 (2.15)	1.53 (1.39)
TC**, mmol/L (*N* = 933,872)		5.14 (1.01)	4.90 (0.95)
HDL-c**, mmol/L (*N* = 923,832)		1.15 (0.29)	1.39 (0.39)
LDL-c**, mmol/L (*N* = 923,715)		2.84 (0.85)	2.72 (0.80)
SBP**, mmHg (*N* = 938,969)		132.81 (16.08)	121.36 (16.31)
DBP**, mmHg (*N* = 938,985)		83.27 (11.57)	74.79 (10.92)
FSG**, mmol/L (*N* = 942,864)		5.84 (1.61)	5.35 (1.18)
Hypertension**	No	62,506 (58.53)	681,807 (81.93)
	Yes	44,278 (41.47)	150,342 (18.07)
Diabetes**	No	94,512 (87.50)	791,011 (94.75)
	Yes	13,500 (12.50)	43,841 (5.25)
Dyslipidemia**	No	40,345 (38.01)	546,177 (66.82)
	Yes	65,785 (61.99)	271,260 (33.18)

** *p <0.01*.

*SD, standard deviation; BMI, body mass index; SBP, systolic blood pressure; DBP, diastolic blood pressure; FSG, fasting serum glucose; TG, triglyceride; TC, total cholesterol; LDL-c, low-density lipoprotein cholesterol; HDL-c, high-density lipoprotein cholesterol*.

*Obesity was defined as BMI ≥ 28 kg/m^2^*.

*Hypertension was defined as self-reported hypertension diagnosed by a physician, self-reported regular use of antihypertensive medications, or systolic/diastolic blood pressure at recruitment ≥ 140/90 mmHg*.

*Dyslipidemia was defined as meeting any of the following criteria: (1) TC ≥ 6.22 mmol/L; (2) LDL-C ≥ 4.14 mmol/L; (3) HDL-C <1.04 mmol/L; (4) TG ≥ 2.26 mmol/L; (5) self-reported dyslipidemia or use of lipid-lowering medications; Diabetes mellitus was defined as self-reported diabetes diagnosed by a physician, self-reported regular use of antidiabetic medications, or fasting glucose at recruitment ≥ 7.0 mmol/L*.

The authors apologize for these errors and state that they do not change the scientific conclusions of the article in any way. The original article has been updated.

## Publisher's Note

All claims expressed in this article are solely those of the authors and do not necessarily represent those of their affiliated organizations, or those of the publisher, the editors and the reviewers. Any product that may be evaluated in this article, or claim that may be made by its manufacturer, is not guaranteed or endorsed by the publisher.

