# Supplementary material for: Trends in Obesity and Metabolic Status in Northern and Southern China Between 2012 and 2020
Source: Front Nutr. 2022 Jan 11;8:811244. doi: 10.3389/fnut.2021.811244 (PMC8786809; doi:10.3389/fnut.2021.811244)
Supplement: Supplementary file 1 [file Data_Sheet_1.PDF]

Supplemental Material

Supplemental file 1: Methods for clinical definition and measurements

Supplemental file 2: Table S1- S6

Supplemental file 3: Figure S1- S7

Supplemental file 1: Methods for clinical definition and measurements

Clinical characteristics

Blood pressure (BP) was measured on the right upper arm in the sitting position after 10-15 min of rest using a validated digital automatic analyzer (Omron 9020). Systolic BP and diastolic BP were each measured twice and the mean of the two readings was considered in the analysis. If the two readings differed by >5mmHg, a third measurement was performed and the average of all three readings was applied.

Data on demographic variables, medical history, lifestyle, and social status (marriage, education, occupation, and annual income) were collected by standardized questionnaires in Hunan. The data collection in Beijing is done by the medical examiner, and the records include: medical history and lifestyle.

Definition of chronic diseases

Hypertension was defined as self-reported hypertension diagnosed by a physician, self-reported regular use of antihypertensive medications, or systolic/diastolic blood pressure at recruitment ≥ 140/90 mmHg.

Dyslipidemia was defined as meeting any of the following criteria: 1) TC≥6.22 mmol/L; 2) LDL-C≥4.14 mmol/L; 3) HDL-C<1.04 mmol/L; 4) TG ≥2.26 mmol/L; 5) self-reported dyslipidemia or use of lipid-lowering medications;

Diabetes mellitus was defined as self-reported diabetes diagnosed by a physician, self-reported regular use of antidiabetic medications, or fasting glucose at recruitment ≥ 7.0 mmol/L.

Laboratory measurements

Fasting venous blood samples were collected and immediately processed and analyzed at the clinical laboratory of Aerospace Center Hospital and Third Xiangya Hospital, respectively. The automated analyzer (Beckman AU5811; Beckman, America) and (Hitachi 7600-110; Hitachi, Japan) were used in Aerospace Center Hospital and Third Xiangya Hospital, respectively.

Fasting blood glucose was measured with the glucose oxidase method. Reagent test kit: DiaSys Diagnostic before July 2019, LEALEADMAN to date in Aerospace Center Hospital; Hitachi in Third Xiangya Hospital. HDL cholesterol and triglycerides were measured with enzymatic

methods. Reagent test kit: DiaSys Diagnostic before July 2019, MC bioengineering to date in Aerospace Center Hospital; Hitachi in Third Xiangya Hospital. The fasting venous blood test data were calibrated when the reagent test kits were changed in Aerospace Center Hospital. All performance of sample analysis was in accordance wih the manufacturer's specifications.

Supplemental file 2: Table S1- S6

Table S1 Mean and 95% confidence interval of BMI levels by age and sex among adults aged 20 years and older in northern and southern China, 2012–2020

Year	Northern China BMI, kg/m^2 (mean [95%CI])			Southern China BMI, kg/m^2 (mean [95%CI])		
	All*	Female	Male	All*	Female	Male
20-39 years old						
2012				22.68(22.64,22.73)	21.10(21.06,21.15)	24.27(24.21,24.32)
2013				22.85(22.82,22.89)	21.21(21.17,21.25)	24.50(24.45,24.55)
2014	23.03(22.94,23.12)	21.60(21.48,21.72)	24.46(24.35,24.57)	22.97(22.94,23.01)	21.35(21.31,21.39)	24.60(24.55,24.64)
2015	22.87(22.80,22.94)	21.41(21.31,21.51)	24.33(24.25,24.41)	23.02(22.98,23.05)	21.43(21.39,21.47)	24.61(24.56,24.66)
2016	23.02(22.96,23.08)	21.62(21.54,21.69)	24.42(24.35,24.49)	22.87(22.83,22.90)	21.30(21.26,21.34)	24.43(24.39,24.48)
2017	23.02(22.96,23.07)	21.56(21.48,21.64)	24.48(24.41,24.54)	22.96(22.93,23.00)	21.43(21.38,21.47)	24.50(24.45,24.55)
2018	23.23(23.19,23.28)	21.80(21.74,21.87)	24.67(24.61,24.73)	23.04(23.00,23.08)	21.41(21.36,21.45)	24.67(24.62,24.72)
2019	23.28(23.24,23.32)	21.83(21.78,21.88)	24.74(24.69,24.79)	23.20(23.16,23.23)	21.58(21.54,21.63)	24.81(24.76,24.86)
2020	23.48(23.43,23.53)	21.85(21.80,21.91)	25.11(25.05,25.16)	23.15(23.11,23.20)	21.51(21.47,21.56)	24.79(24.73,24.85)
p for overall	<0.001	<0.001	<0.001	<0.001	<0.001	<0.001
p for nonlinear trend	<0.001	0.685	<0.001	0.979	0.013	0.060

Subtotal	23.21(23.19,23.23)	21.74(21.72,21.77)	24.67(24.65,24.70)	22.97(22.96,22.98)	21.37(21.36,21.39)	24.57(24.55,24.59)
40- 59 years old						
2012				24.09(24.05,24.12)	23.05(22.99,23.10)	25.13(25.09,25.17)
2013				24.26(24.23,24.29)	23.18(23.13,23.23)	25.34(25.30,25.38)
2014	24.37(24.29,24.46)	23.22(23.10,23.34)	25.53(25.42,25.64)	24.37(24.34,24.40)	23.36(23.31,23.40)	25.38(25.34,25.42)
2015	24.19(24.12,24.25)	23.12(23.02,23.21)	25.25(25.17,25.34)	24.34(24.30,24.37)	23.27(23.23,23.32)	25.40(25.36,25.44)
2016	24.29(24.23,24.35)	23.17(23.08,23.25)	25.41(25.33,25.49)	24.20(24.16,24.23)	23.15(23.11,23.20)	25.24(25.20,25.28)
2017	24.34(24.28,24.40)	23.20(23.11,23.29)	25.48(25.41,25.55)	24.28(24.25,24.31)	23.23(23.19,23.28)	25.33(25.29,25.37)
2018	24.50(24.45,24.56)	23.46(23.38,23.53)	25.55(25.48,25.61)	24.23(24.20,24.27)	23.11(23.06,23.15)	25.36(25.32,25.40)
2019	24.62(24.58,24.67)	23.60(23.54,23.67)	25.64(25.58,25.70)	24.29(24.26,24.33)	23.20(23.15,23.24)	25.39(25.35,25.43)
2020	24.70(24.64,24.75)	23.59(23.51,23.66)	25.81(25.74,25.88)	24.21(24.17,24.25)	23.04(22.99,23.09)	25.38(25.33,25.43)
p for overall trend	<0.001	<0.001	<0.001	<0.001	<0.001	<0.001
p for nonlinear trend	<0.001	0.036	<0.001	<0.001	<0.001	0.024
Subtotal	24.47(24.45,24.49)	23.39(23.35,23.42)	25.55(25.52,25.58)	24.26(24.25,24.27)	23.19(23.17,23.20)	25.33(25.32,25.34)
60- years old						
2012				24.03(23.96,24.10)	23.84(23.72,23.95)	24.23(24.15,24.30)
2013				24.21(24.15,24.27)	24.02(23.93,24.12)	24.40(24.33,24.47)

2014	24.74(24.66,24.82)	24.67(24.55,24.79)	24.81(24.71,24.92)	24.28(24.22,24.33)	24.06(23.97,24.15)	24.49(24.43,24.56)
2015	24.55(24.48,24.62)	24.40(24.31,24.50)	24.69(24.60,24.78)	24.28(24.23,24.33)	24.01(23.93,24.10)	24.55(24.48,24.61)
2016	24.55(24.48,24.62)	24.42(24.32,24.52)	24.69(24.59,24.78)	24.16(24.10,24.21)	23.85(23.77,23.94)	24.46(24.40,24.52)
2017	24.48(24.41,24.54)	24.34(24.24,24.43)	24.62(24.53,24.71)	24.15(24.09,24.20)	23.79(23.70,23.88)	24.50(24.44,24.57)
2018	24.63(24.56,24.69)	24.53(24.43,24.62)	24.73(24.64,24.82)	24.03(23.97,24.10)	23.67(23.57,23.77)	24.40(24.32,24.47)
2019	24.67(24.61,24.73)	24.54(24.46,24.63)	24.79(24.71,24.88)	24.16(24.10,24.22)	23.82(23.72,23.92)	24.50(24.42,24.58)
2020	24.67(24.59,24.75)	24.47(24.35,24.58)	24.87(24.76,24.97)	24.09(24.02,24.17)	23.72(23.60,23.84)	24.47(24.37,24.56)
p for overall	<0.001	0.034	0.002	<0.001	<0.001	<0.001
p for nonlinear trend	<0.001	0.012	<0.001	<0.001	0.103	<0.001
Subtotal	24.61(24.58,24.63)	24.48(24.44,24.51)	24.73(24.70,24.77)	24.16(24.14,24.18)	23.88(23.85,23.91)	24.45(24.42,24.47)

* Estimates are adjusted by sex; Abbreviation: BMI, body mass index.

Table S2 Age- standardized mean and 95% confidence interval of fasting serum glucose levels among adults aged 20 years and older in northern and southern China, 2012–2020

Year	Northern China FSG, mmol/L (mean [95%CI])			Southern China FSG, mmol/L (mean [95%CI])		
	All$	Female	Male	All$	Female	Male
2012				5.33(5.32,5.34)	5.21(5.20,5.22)	5.46(5.45,5.47)
2013				5.31(5.30,5.32)	5.17(5.16,5.18)	5.44(5.43,5.45)
2014	5.23(5.22,5.25)	5.11(5.10,5.13)	5.35(5.33,5.38)	5.33(5.32,5.34)	5.21(5.20,5.22)	5.46(5.44,5.47)
2015	5.29(5.28,5.31)	5.17(5.16,5.19)	5.41(5.39,5.43)	5.33(5.32,5.34)	5.20(5.19,5.21)	5.46(5.45,5.47)
2016	5.37(5.36,5.38)	5.24(5.23,5.25)	5.50(5.48,5.52)	5.40(5.40,5.41)	5.26(5.25,5.27)	5.55(5.53,5.56)
2017	5.32(5.30,5.33)	5.19(5.17,5.21)	5.44(5.42,5.46)	5.47(5.46,5.48)	5.33(5.32,5.34)	5.60(5.59,5.62)
2018	5.33(5.31,5.34)	5.21(5.19,5.22)	5.44(5.43,5.46)	5.53(5.52,5.54)	5.36(5.35,5.38)	5.70(5.68,5.71)
2019	5.27(5.26,5.28)	5.14(5.13,5.16)	5.40(5.39,5.42)	5.56(5.55,5.57)	5.41(5.39,5.42)	5.71(5.70,5.73)
2020	5.38(5.36,5.39)	5.25(5.23,5.26)	5.51(5.49,5.53)	5.46(5.45,5.47)	5.32(5.31,5.34)	5.60(5.58,5.62)
p for overall	<0.001	<0.001	<0.001	<0.001	<0.001#	<0.001
p for nonlinear trend	0.014	0.041	0.007	0.013	0.059	0.039
Subtotal	5.32(5.31,5.32)	5.19(5.19,5.20)	5.44(5.44,5.45)	5.41(5.41,5.41)	5.27(5.27,5.28)	5.55(5.54,5.55)

* Estimates are age-standardized to the 2010 Chinese Census population using age groups 20-29, 30-39, 40-49, 50-59, 60-69 and 70 or older; $ additional adjusted by sex; # *p* for linear trend<0.001; Abbreviation: FSG, fasting serum glucose.

Table S3 Age-standardized mean and 95% confidence interval of triglyceride levels among adults aged 20 years and older in northern and southern China, 2012–2020

Year	Northern China TG, mmol/L (mean [95%CI])			Southern China TG, mmol/L (mean [95%CI])		
	All$	Female	Male	All$	Female	Male
2012				1.58(1.57,1.59)	1.19(1.18,1.20)	1.97(1.95,1.98)
2013				1.64(1.63,1.65)	1.24(1.23,1.25)	2.05(2.03,2.06)
2014	1.46(1.44,1.48)	1.19(1.17,1.21)	1.73(1.70,1.76)	1.57(1.56,1.58)	1.19(1.18,1.20)	1.95(1.94,1.97)
2015	1.37(1.36,1.39)	1.13(1.11,1.14)	1.62(1.59,1.64)	1.58(1.57,1.59)	1.19(1.18,1.20)	1.96(1.94,1.98)
2016	1.41(1.39,1.42)	1.15(1.14,1.16)	1.67(1.65,1.69)	1.67(1.66,1.68)	1.28(1.27,1.29)	2.06(2.05,2.08)
2017	1.39(1.38,1.41)	1.14(1.13,1.16)	1.64(1.62,1.66)	1.69(1.68,1.70)	1.32(1.31,1.33)	2.06(2.04,2.07)
2018	1.43(1.41,1.44)	1.17(1.16,1.18)	1.68(1.66,1.70)	1.67(1.66,1.68)	1.25(1.24,1.26)	2.09(2.07,2.11)
2019	1.44(1.43,1.45)	1.19(1.18,1.20)	1.68(1.67,1.70)	1.67(1.66,1.68)	1.25(1.24,1.26)	2.08(2.06,2.10)
2020	1.45(1.44,1.47)	1.20(1.19,1.22)	1.71(1.69,1.72)	1.75(1.74,1.77)	1.30(1.28,1.31)	2.21(2.18,2.23)
p for overall	<0.001	<0.001	<0.001	<0.001	<0.001	<0.001
p for nonlinear trend	<0.001	<0.001	<0.001	<0.001	<0.001	<0.001
Subtotal	1.42(1.42,1.43)	1.17(1.17,1.18)	1.68(1.67,1.68)	1.64(1.64,1.65)	1.25(1.24,1.25)	2.04(2.03,2.05)

* Estimates are age-standardized to the 2010 Chinese Census population using age groups 20-29, 30-39, 40-49, 50-59, 60-69 and 70 or older;
$ additional adjusted by sex; Abbreviation: TG, triglyceride.

Table S4 Age-standardized mean and 95% confidence interval of high-density lipoprotein cholesterol levels among adults aged 20 years and older in northern and southern China, 2012–2020

Year	Northern China HDL-c, mmol/L (mean [95%CI])			Southern China HDL-c, mmol/L (mean [95%CI])		
	All$	Female	Male	All$	Female	Male
2012				1.61(1.60,1.61)	1.78(1.78,1.79)	1.43(1.43,1.43)
2013				1.62(1.62,1.62)	1.80(1.80,1.81)	1.44(1.43,1.44)
2014	0.92(0.92,0.92)	1.00(0.99,1.00)	0.84(0.84,0.84)	1.64(1.64,1.64)	1.83(1.82,1.83)	1.46(1.45,1.46)
2015	0.93(0.93,0.93)	1.01(1.00,1.01)	0.86(0.86,0.86)	1.54(1.54,1.55)	1.71(1.70,1.71)	1.38(1.38,1.38)
2016	0.96(0.96,0.96)	1.04(1.04,1.05)	0.88(0.88,0.88)	1.36(1.35,1.36)	1.49(1.49,1.49)	1.22(1.22,1.22)
2017	0.96(0.95,0.96)	1.04(1.04,1.04)	0.87(0.87,0.87)	1.44(1.44,1.44)	1.57(1.57,1.57)	1.31(1.31,1.31)
2018	1.28(1.28,1.29)	1.40(1.39,1.40)	1.17(1.16,1.17)	1.38(1.37,1.38)	1.52(1.51,1.52)	1.24(1.23,1.24)
2019	1.25(1.25,1.25)	1.37(1.36,1.37)	1.14(1.13,1.14)	1.35(1.34,1.35)	1.47(1.47,1.48)	1.22(1.22,1.22)
2020	1.30(1.29,1.30)	1.41(1.41,1.42)	1.18(1.17,1.18)	1.33(1.33,1.33)	1.45(1.45,1.46)	1.20(1.20,1.21)
p for overall	<0.001	<0.001	<0.001	<0.001	<0.001	<0.001
p for nonlinear trend	<0.001	<0.001	<0.001	<0.001	<0.001	<0.001
Subtotal	1.12(1.12,1.12)	1.22(1.22,1.23)	1.01(1.01,1.01)	1.48(1.48,1.48)	1.63(1.62,1.63)	1.33(1.33,1.33)

* Estimates are age-standardized to the 2010 Chinese Census population using age groups 20-29, 30-39, 40-49, 50-59, 60-69 and 70 or older;
$ additional adjusted by sex; Abbreviation: HDL-C, high-density lipoprotein cholesterol.

Table S5 Age-standardized mean and 95% confidence interval of systolic blood pressure levels among adults aged 20 years and older in northern and southern China, 2012–2020

Year	Northern China SBP, mmol/L (mean [95%CI])			Southern China SBP, mmol/L (mean [95%CI])		
	All$	Female	Male	All$	Female	Male
2012				121.51(121.38,121.64)	117.09(116.90,117.28)	125.93(125.78,126.07)
2013				121.73(121.62,121.84)	117.03(116.86,117.19)	126.43(126.30,126.56)
2014	119.51(119.28,119.75)	116.05(115.72,116.37)	122.98(122.70,123.27)	121.93(121.82,122.03)	117.68(117.53,117.83)	126.17(126.05,126.29)
2015	121.38(121.19,121.56)	117.83(117.57,118.09)	124.93(124.71,125.15)	122.14(122.04,122.25)	117.72(117.57,117.86)	126.57(126.45,126.70)
2016	122.74(122.57,122.90)	119.35(119.12,119.58)	126.12(125.91,126.33)	120.93(120.82,121.03)	116.51(116.36,116.66)	125.35(125.22,125.47)
2017	122.38(122.20,122.55)	118.63(118.39,118.87)	126.12(125.91,126.33)	121.25(121.15,121.36)	116.73(116.57,116.88)	125.78(125.65,125.91)
2018	123.24(123.08,123.40)	119.93(119.71,120.16)	126.55(126.35,126.75)	121.71(121.60,121.82)	117.33(117.17,117.49)	126.09(125.96,126.23)
2019	123.99(123.84,124.15)	120.64(120.42,120.86)	127.35(127.15,127.54)	122.69(122.58,122.80)	118.49(118.32,118.65)	126.89(126.76,127.03)
2020	125.21(125.04,125.38)	121.50(121.26,121.74)	128.91(128.69,129.14)	122.74(122.61,122.87)	119.04(118.85,119.23)	126.43(126.27,126.60)
p for overall	<0.001	<0.001	<0.001#	<0.001	<0.001	<0.001
p for nonlinear trend	<0.001	<0.001	0.077	<0.001	<0.001	<0.001
Subtotal	122.99(122.93,123.06)	119.53(119.44,119.63)	126.45(126.37,126.54)	121.82(121.78,121.85)	117.47(117.41,117.52)	126.17(126.12,126.21)

* Estimates are age-standardized to the 2010 Chinese Census population using age groups 20-29, 30-39, 40-49, 50-59, 60-69 and 70 or older;
$ additional adjusted by sex; # *p* for linear trend<0.001; Abbreviation: SBP, systolic blood pressure.

Table S6 Age-standardized mean and 95% confidence interval of diastolic blood pressure levels among adults aged 20 years and older in northern and southern China, 2012–2020

Year	Northern China DBP, mmol/L (mean [95%CI])			Southern China DBP, mmol/L (mean [95%CI])		
	All[$]	Female	Male	All[$]	Female	Male
2012				74.45(74.36,74.54)	70.95(70.82,71.07)	77.95(77.84,78.06)
2013				74.26(74.18,74.33)	70.55(70.44,70.66)	77.97(77.87,78.06)
2014	75.31(75.14,75.47)	72.57(72.34,72.80)	78.04(77.83,78.25)	74.91(74.84,74.99)	71.44(71.34,71.55)	78.38(78.29,78.47)
2015	76.70(76.57,76.83)	73.88(73.70,74.06)	79.52(79.36,79.68)	74.83(74.75,74.90)	71.27(71.17,71.37)	78.39(78.29,78.48)
2016	77.12(77.01,77.24)	74.12(73.96,74.28)	80.13(79.98,80.28)	74.07(74.00,74.14)	70.46(70.36,70.56)	77.69(77.60,77.78)
2017	78.09(77.96,78.21)	75.00(74.83,75.18)	81.17(81.01,81.33)	73.83(73.76,73.91)	70.12(70.01,70.22)	77.55(77.45,77.64)
2018	78.73(78.61,78.85)	75.73(75.56,75.89)	81.73(81.58,81.88)	73.63(73.55,73.71)	69.89(69.78,70.00)	77.38(77.28,77.48)
2019	78.59(78.48,78.70)	75.23(75.08,75.39)	81.95(81.81,82.10)	74.27(74.19,74.34)	70.64(70.53,70.75)	77.89(77.79,77.99)
2020	78.24(78.12,78.36)	74.67(74.51,74.84)	81.81(81.65,81.96)	74.82(74.73,74.91)	71.65(71.52,71.77)	77.99(77.87,78.11)
p for overall	<0.001	<0.001	<0.001	<0.001	<0.001	<0.001
p for nonlinear trend	<0.001	<0.001	<0.001	<0.001	<0.001	0.014
Subtotal	77.79(77.74,77.83)	74.66(74.60,74.72)	80.91(80.85,80.97)	74.33(74.30,74.35)	70.75(70.71,70.78)	77.91(77.88,77.94)

* Estimates are age-standardized to the 2010 Chinese Census population using age groups 20-29, 30-39, 40-49, 50-59, 60-69 and 70 or older;
$ additional adjusted by sex; Abbreviation: DBP, diastolic blood pressure.

Supplemental file 3: Figure S1- S7

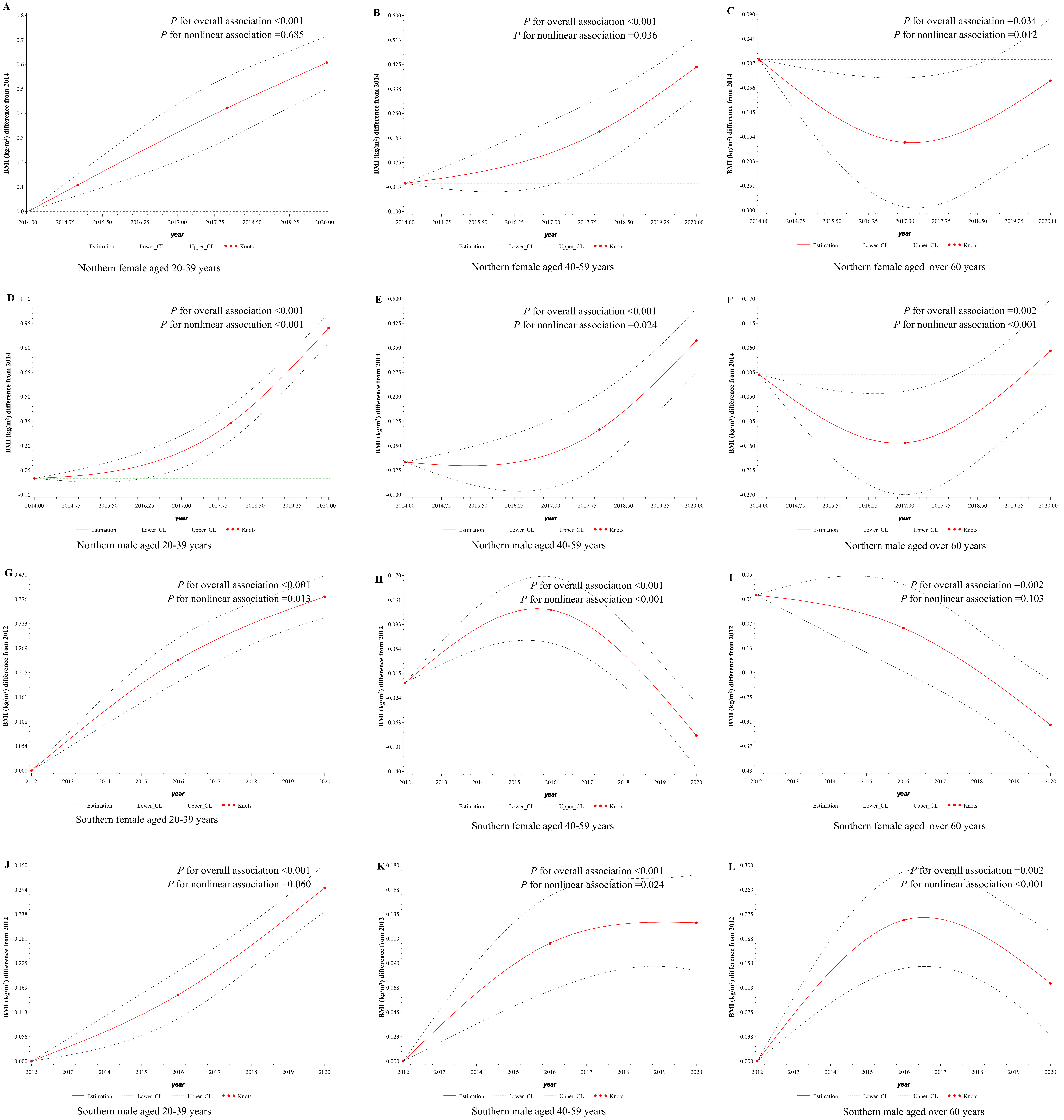

Figure S1. Temporal trends of BMI among different aged and gender groups in northern and southern adult Chinese during 2012-2020*

*Surveyed year was coded using an RCS function with three knots located at the 5th, 50th, and 95th percentiles of the distribution of survey years. *Y*-axis represents the BMI difference from referenced year. Referenced year in (A) to (F) was 2014 and in (G) to (L) was 2012. Dashed lines are 95 per cent confidence intervals. Knots are represented by dots.

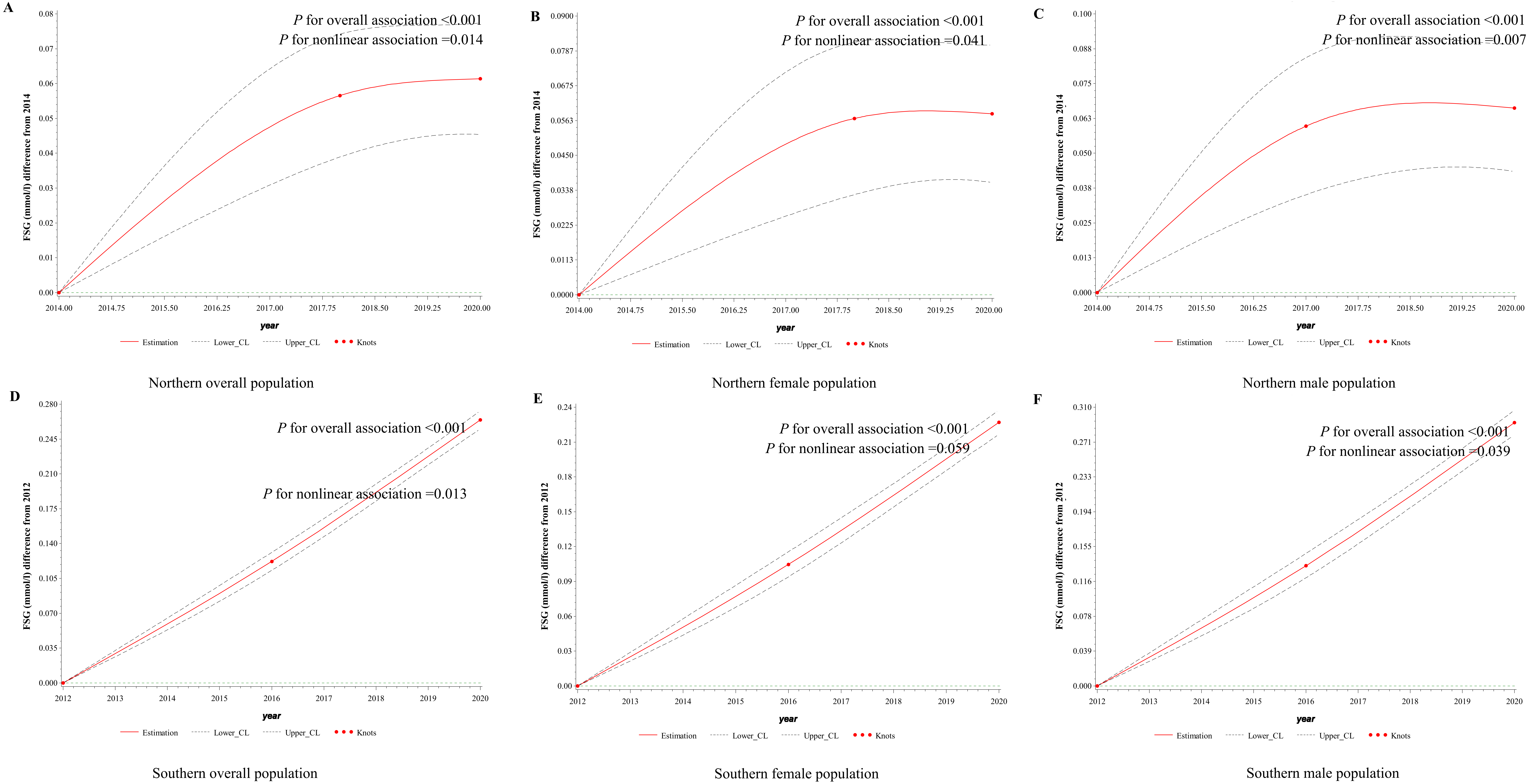

Figure S2. Temporal trends of FSG among different aged and gender groups in northern and southern adult Chinese during 2012-2020

*Surveyed year was coded using an RCS function with three knots located at the 5th, 50th, and 95th percentiles of the distribution of survey years. *Y*-axis represents the FSG difference from referenced year. Referenced year in (A) to (C) was 2014 and in (D) to (F) was 2012. Dashed lines are 95 per cent confidence intervals. Knots are represented by dots. In (A) and (D), age and sex were included as adjustment variables.; In (B), (C), (E) and (F), age was included as adjustment variable.

Abbreviation: FSG, Fasting Serum Glucose

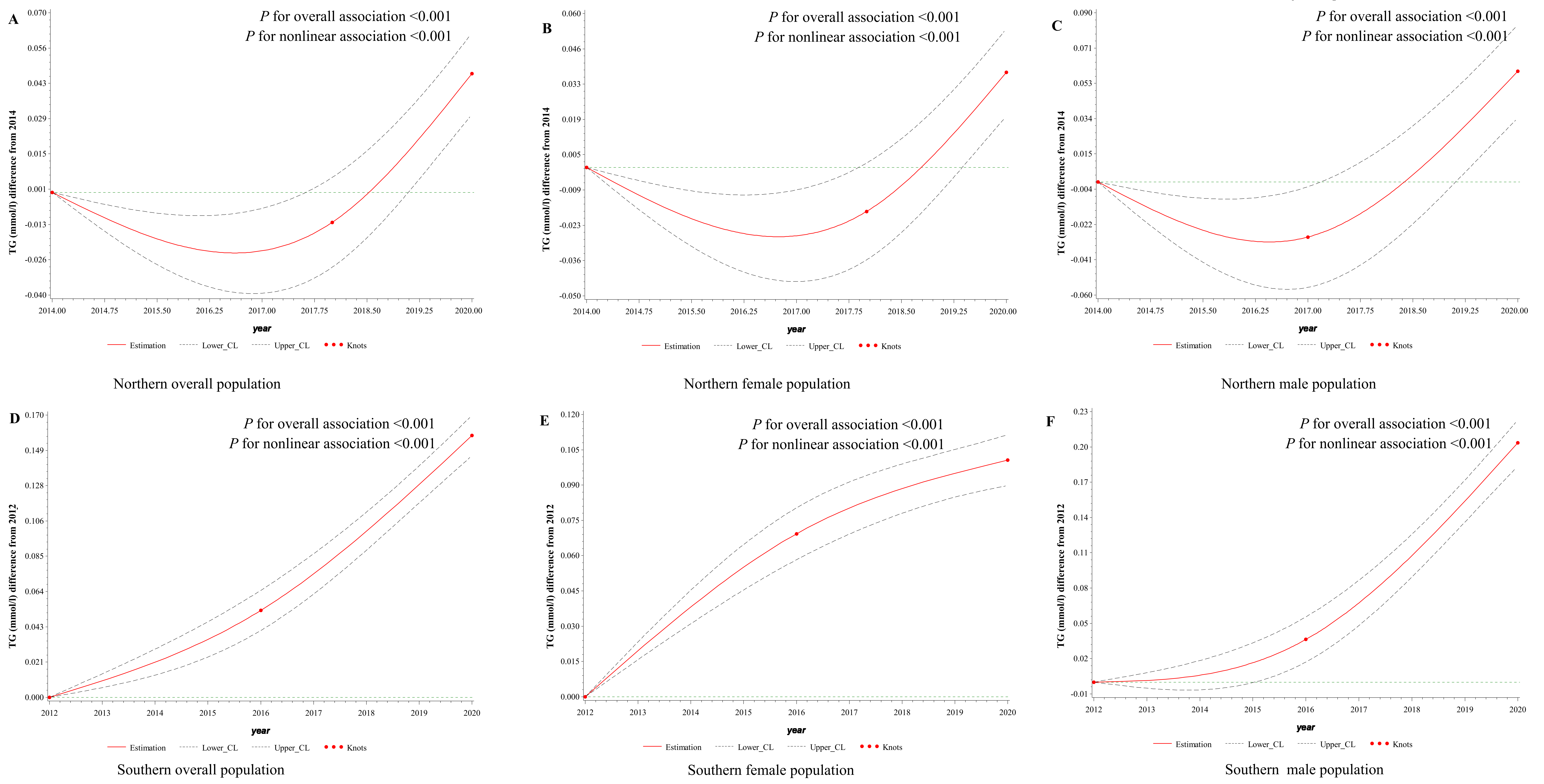

Figure S3. Temporal trends of Triglycride among among different aged and gender groups in northern and southern adult Chinese during 2012-2020

*Surveyed year was coded using an RCS function with three knots located at the 5th, 50th, and 95th percentiles of the distribution of survey years. *Y*-axis represents the TG difference from referenced year. Referenced year in (A) to (C) was 2014 and in (D) to (F) was 2012. Dashed lines are 95 per cent confidence intervals. Knots are represented by dots. In (A) and (D), age and sex were included as adjustment variables.; In (B), (C), (E) and (F), age was included as adjustment variable.

Abbreviation: TG, Triglycride

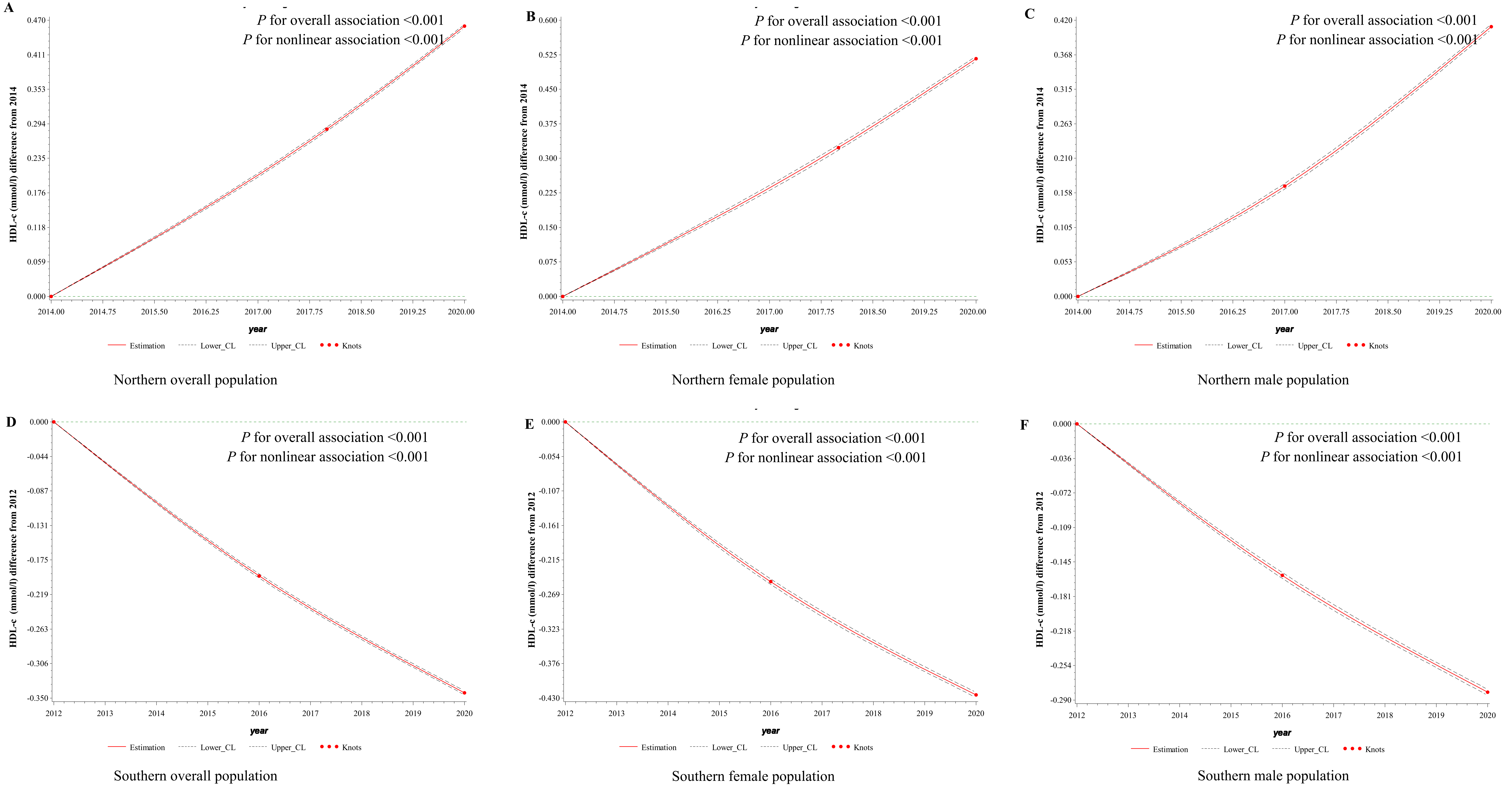

Figure S4. Temporal trends of HDL-c among different aged and gender groups in northern and southern adult Chinese during 2012-2020

*Surveyed year was coded using an RCS function with three knots located at the 5th, 50th, and 95th percentiles of the distribution of survey years. *Y*-axis represents the HDL-c difference from referenced year. Referenced year in (A) to (C) was 2014 and in (D) to (F) was 2012. Dashed lines are 95 per cent confidence intervals. Knots are represented by dots. In (A) and (D), age and sex were included as adjustment variables.; In (B), (C), (E) and (F), age was included as adjustment variable.

Abbreviation: HDL-c, High-Density Lipoprotein cholesterol

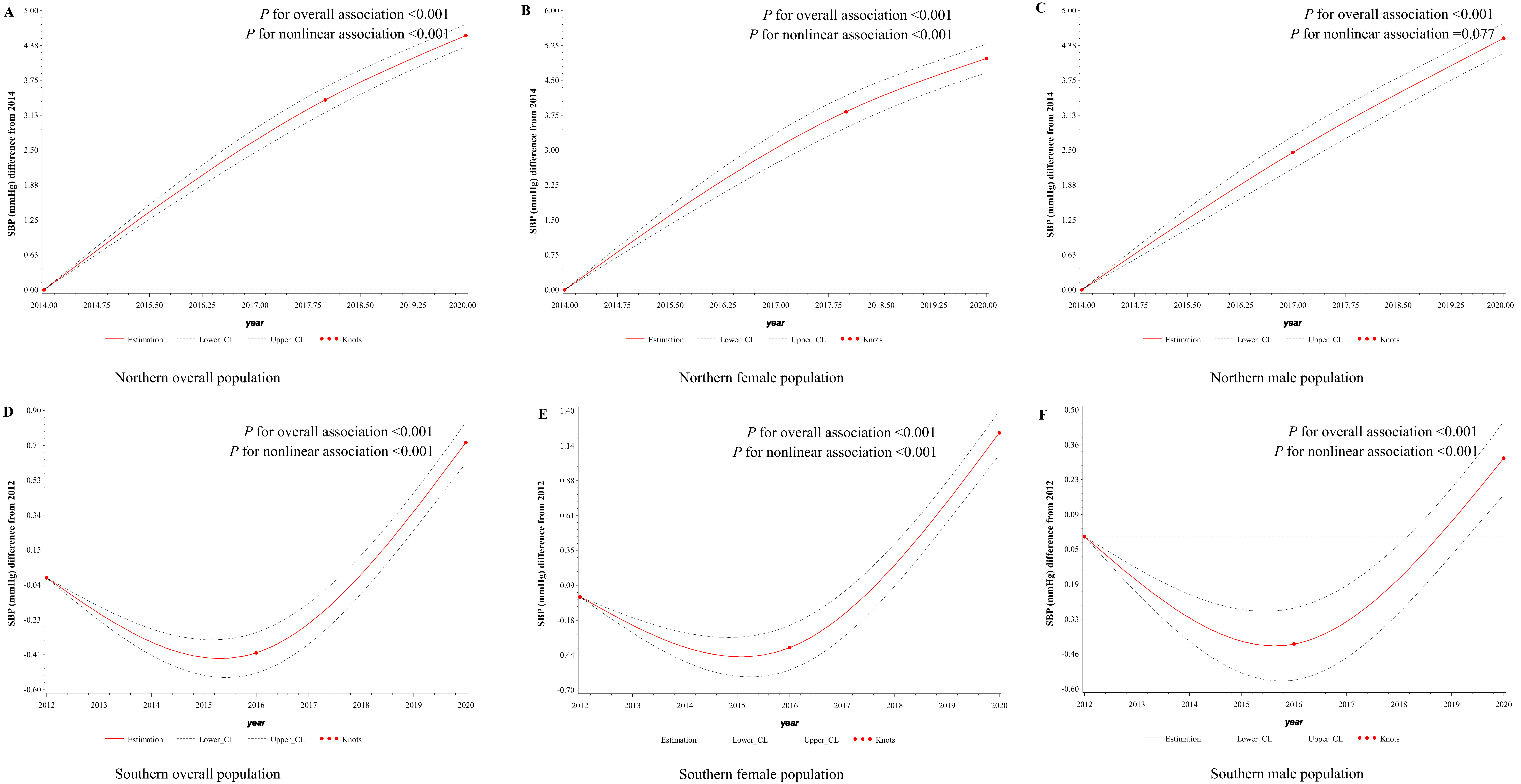

Figure S5. Temporal trends of SBP among different aged and gender groups in northern and southern adult Chinese during 2012-2020

*Surveyed year was coded using an RCS function with three knots located at the 5th, 50th, and 95th percentiles of the distribution of survey years. *Y*-axis represents the SBP difference from referenced year. Referenced year in (A) to (C) was 2014 and in (D) to (F) was 2012. Dashed lines are 95 per cent confidence intervals. Knots are represented by dots. In (A) and (D), age and sex were included as adjustment variables.; In (B), (C), (E) and (F), age was included as adjustment variable.
Abbreviation: SBP, Systolic Blood Pressure

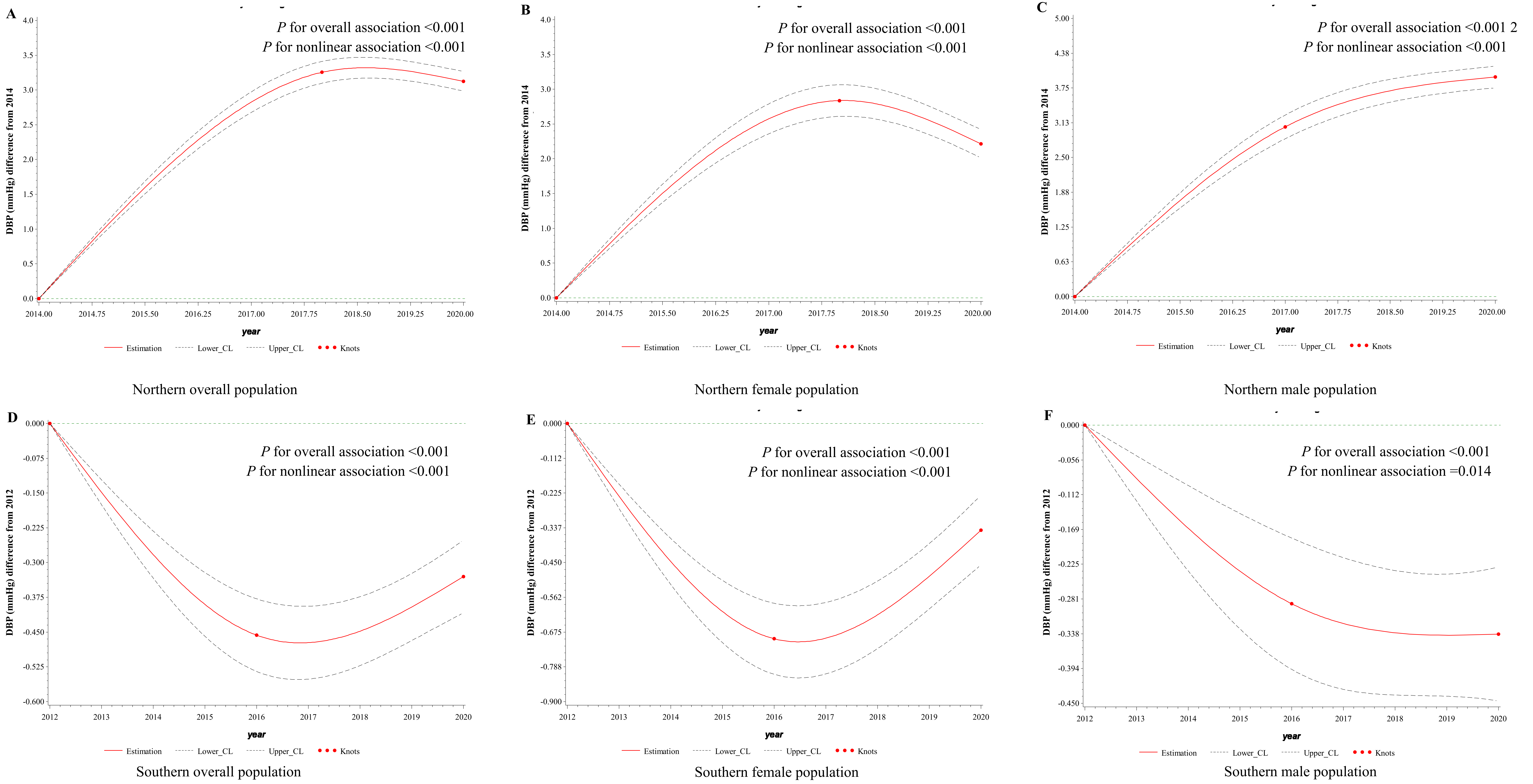

Figure S6. Temporal trends of DBP among different aged and gender groups in northern and southern adult Chinese during 2012-2020

*Surveyed year was coded using an RCS function with three knots located at the 5th, 50th, and 95th percentiles of the distribution of survey years. *Y*-axis represents the DBP difference from referenced year. Referenced year in (A) to (C) was 2014 and in (D) to (F) was 2012. Dashed lines are 95 per cent confidence intervals. Knots are represented by dots. In (A) and (D), age and sex were included as adjustment variables.; In (B), (C), (E) and (F), age was included as adjustment variable.

Abbreviation: DBP, Diastolic Blood Pressure

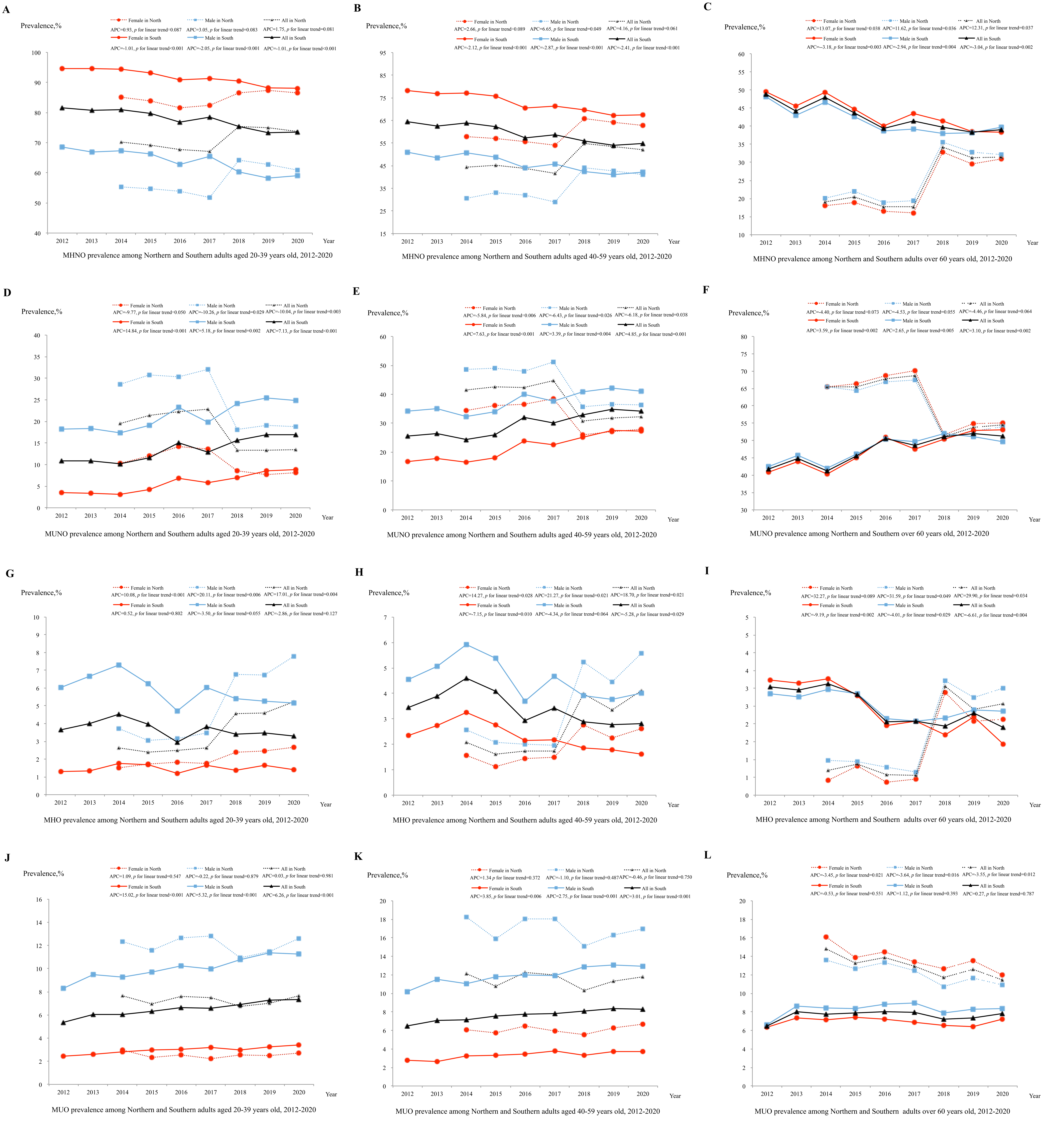

Figure S7 Trend in prevalence of obesity by metabolic status phenotype in different age and gender groups in northern and southern adult Chinese from 2012 to 2020*

*Estimates for overall population are standardized by gender; Significant linear trends (P <0.05) for the following groups :(1) decreased MHNO among all aged and gender groups from southern China; (2) increased MHNO among 40-59 aged male group and 60 and over aged group from northern China; (3) decreased MUNO among 20-39 aged group and 40-59 aged female from northern China; (4) increased MUNO among all aged and gender groups from southern China; (5) increased MHO among all 20-39 and 40-59 aged groups, and 60 and over aged male from northern China; (6) decreased MHO among 40-59 aged female and all 60 and over aged groups from southern China(P<0.05); (7) decreased MUO among 60 and over aged groups from northern China; (8)increased MUO among all 20-39 and 40-59 aged groups from southern China.

Abbreviation: MHNO, metabolic healthy non-obese; MUNO, metabolic unhealthy non-obese; MHO, metabolic healthy obese; MUO: metabolic unhealthy obese; APC, annual percentage change
